# Psychological management for head and neck cancer patients: United Kingdom National Multidisciplinary Guidelines

**DOI:** 10.1017/S0022215116000426

**Published:** 2016-05

**Authors:** G Humphris

**Affiliations:** University of St Andrews, Medical School, North Haugh, St Andrews, Fife, UK

## Abstract

This is the official guideline endorsed by the specialty associations involved in the care of head and neck cancer patients in the UK. It provides recommendations on the assessment and interventions for the psychological management in this patient group.

**Recommendations:**

• Audit of information supplied to patients and carers should be conducted on an annual basis to update and review content and media presentation. (G)

• Patients and carers should be invited to discuss treatment options and relate possible outcomes to functional retention or loss to provide a patient-centred approach. (G)

• Clinical staff should inspect their systems of assessment to make them sensitive enough to identify patients with psychological difficulties. (G)

• Flexibility, rather than rigid formulation is required to assess patients frequently, and to allow for change in circumstances to be noted. (G)

• Multidisciplinary teams should determine the supportive care services available and commission extra assistance to provide patients and carers with timely information, education or brief supportive advice. (G)

• Multidisciplinary teams need to inspect specialist services for mental health interventions at structured and complex levels for the small proportion of patients with more serious, but rarer, psychological difficulties. (G)

• Clinical staff at all levels should receive communication skills training to raise and maintain consultation expertise with difficult patient and/or carer interactions. (G)

## Introduction

The head and neck cancer patient and their carers have considerable challenges to overcome.^1^ The psychological experience of the patient with head and neck cancer has been closely described in a recent systematic review and meta-synthesis.^2^ Although many patients appear to cope surprisingly well, a sizeable minority experience considerable psychological effects including uncertainty about the return of cancer, disruption to daily life, a diminished self, attempts to understand the changes that occur and finding a plan forward. Treatment recovery may be hampered by mood changes, whereas longer term psychological states may feature some months and even years following initial treatment.^3^ This section has benefited from recent research in the field and highlights the major psychological management concerns in the course of caring for the patient being treated for head and neck cancer.^4^

## Communication of diagnosis and treatment

Evidence from other areas of treating cancer at other sites has demonstrated clearly that the way in which the diagnosis is presented to the patient is important to their psychological response to the disease and treatment.^5^^,^^6^ It is vital that the patient is told explicitly that they have a cancer, its nature and that all treatment available is presented to them in an unambiguous manner. This needs to be relayed consistently by all members of the team, so that the patient and carer are able to draw upon their coping abilities as well as possible. Recent evidence shows that delivering information without interruption, avoiding jargon and showing appropriate empathy are important features of the diagnostic interview to help prevent illness concerns developing.^6^ Decision-making and designing tools to improve communication between clinician and patient is improving rapidly and highlights an important growth area for the future of head and neck cancer care where complex choices are discussed and commitments made with patients.^7^^,^^8^

## Delivering information about treatment and recovery

Considerable efforts have been expended to determine the information needs of head and neck cancer patients.^9^^,^^10^ Poor satisfaction with information supplied by the team was predictive of patient lowered mood and quality of life (QoL) in the longer term.^11^ More information was required on financial advice, support groups and ability to return to work. Virtually no studies have been reported on patient desire to be involved in treatment decision making. The nature of the disease and its complex profile of mixed treatment methods have favoured the multidisciplinary team's (MDT) sole authority to determine treatment regimens. However, recent reports have compiled large datasets of ‘normative’ QoL estimates linked to various treatment options, which enable the team to start sharing the potential risks and benefits of certain treatment packages and tailoring to patient preferences of retained functions on recovery.^12^

**Recommendations**

• Audit of information supplied to patients and carers should be conducted on an annual basis to update and review content and media presentation (G)
• Patients and carers should be invited to discuss treatment options and relate possible outcomes to functional retention or loss to provide a patient-centred approach (G)

## Managing psychological distress

The use of routine assessments for psychological distress such as the Distress Thermometer and the Hospital Anxiety and Depression Scale are being considered as a means to identify those patients who may suffer during the process of treatment preparation, the treatment itself, initial stages of recovery and follow-up out-patient appointments.^13^ These assessments have the ability to capture those patients who would not necessarily be identified by the MDT as needing psychological support.^14^ Two issues follow however: an increased number of patients in need of assistance; and screening measures that may indicate substantial distress when there is none due to measurement error.

The types of psychological distress require attention and definition. The classical typology of mental distress includes anxiety and depression. In addition, assessments of recurrence fears (the most frequent reported concern of head and neck cancer patients), facial disfigurement, body image, loneliness and sexual dysfunction may also be compiled within an MDT assessment profile library for occasional use when required.^15^^,^^16^ Recurrence fears have been found to be linked closely to depression in patients and some evidence exists that patients can stimulate these fears in their carers.^17^ Furthermore, it is now recognised that high recurrence fears promote more requests for medical services incurring higher treatment and surveillance costs. Acknowledgement of the patient experience of the severity and longevity of these fears is important and more in-depth approaches may be required to alleviate debilitating distress.^18^

The profile of staff expertise and skills needs close inspection to enable a flexible and tailored matching of need to professional training of support or specialist staff. Multidisciplinary teams need to plan their services to provide escalating level of care according to the specific need of psychological difficulty presented by the patient. The newly developing Map of Medicine describes in detail the levels of intervention (1–4). Timely support and educational approaches are conducted at levels 1 and 2. Structured interventions are provided at level 3 by staff with a mental health qualification. Level 4 interventions consisting of complex psychotherapeutic approaches are delivered by clinical psychologists, counselling psychotherapists and liaison psychiatrists.

**Recommendations**

• Clinical staff should inspect their systems of assessment to make them sensitive enough to identify patients with psychological difficulties (G)
• Flexibility, rather than rigid formulation is required to assess patients frequently, and to allow for change in circumstances to be noted (G)
• Multidisciplinary teams should determine the supportive care services available and commission extra assistance to provide patients and carers with timely information, education or brief supportive advice (G)
• Multidisciplinary teams need to inspect specialist services for mental health interventions at structured and complex levels for the small proportion of patients with more serious, but rarer, psychological difficulties (G)

## Family and social support

It is important for the MDT to raise survivorship issues with patients.^19^ Not only does the patient remain watchful for indicators and symptoms that may raise concern for life reducing disease processes, but also to maintain function for as long as possible. Two areas are pertinent here. Firstly carers and spouses should be encouraged to use techniques to enhance adherence of follow-up MDT recommendations. Second and closely related is the use of social media to link other members of the local community with similar health conditions and survivorship concerns who can share information and provide extended social support outside the hospital boundaries.

## End of life issues

Communication with the patient assumes even greater importance when curative treatment options are not available and care focuses towards a palliative approach.^20^ Areas such as assessing patient preferences concerning life expectancy, control of pain, and managing fears of uncertainty and family reactions are features of these discussions with the staff of the MDT and palliative care services. The psychological burden to staff requires recognition, supervision and training.

**Recommendation**

• Clinical staff at all levels should receive communication skills training to raise and maintain consultation expertise with difficult patient and/or carer interactions (G)

### Key points

• Develop information services for patients and carers. Consider introducing new technology to collect routine patient self-report data on health behaviour, psychological responses to care received, outlining of key messages and outcome assessments
• Develop decision-making tools (such as explanatory tablet applications) for the aid of patients to enter into discussion with multidisciplinary team to agree on treatment plan
• Collect routine psychological assessments at key points during course of care. These indicators must be supported with dedicated and tailored interventions to prevent neglect of identified psychological distress or depression
• Focus on level of support and intervention that current team can realistically provide with current level of resource. Remain cautious when introducing change, but strengthen and build upon supports already available
• Develop more comprehensive support services by improving generic communication skills training for current staff and ensure consistency of message giving to patients and/or carers across the multidisciplinary team
• Introduce staff training to assist with management of potential burnout in multidisciplinary team staff. Consider flexible responses including secondments, study breaks and peer-support programmes
• Audit current psychological services applied in the head and neck cancer service. Identify current usage, gaps in service and develop forward plans to address these gaps
• Assess current capability of specialist clinical nurse skills to support head and neck cancer patients psychologically, and introduce dedicated training and supervision programmes
• Actively search for clinical psychology service input and negotiate improved access and response time. Estimate likely demand of service
• Consider appointing sessional input to cancer network of a clinical or counselling psychologist or psychotherapist
• Identify liaison psychiatry service and negotiate referral pathway and response time.
